# A signature of seven immune‐related genes predicts overall survival in male gastric cancer patients

**DOI:** 10.1186/s12935-021-01823-0

**Published:** 2021-02-18

**Authors:** Xin Xu, Yida Lu, Youliang Wu, Mingliang Wang, Xiaodong Wang, Huizhen Wang, Bo Chen, Yongxiang Li

**Affiliations:** 1 Department of General Surgery, The First Affiliated Hospital of Anhui Medical University, 218 JiXi Avenue, Hefei, 230022 Anhui China; 2 Anhui Medical University, Hefei, 230022 China

**Keywords:** Immune‐related genes, Prognosis, The Cancer Genome Atlas, Gastric cancer, Male, Overall survival

## Abstract

**Background:**

Gastric cancer (GC) has a high mortality rate and is one of the most fatal malignant tumours. Male sex has been proven as an independent risk factor for GC. This study aimed to identify immune-related genes (IRGs) associated with the prognosis of male GC.

**Methods:**

RNA sequencing and clinical data were obtained from The Cancer Genome Atlas (TCGA) database. Differentially expressed IRGs between male GC and normal tissues were identified by integrated bioinformatics analysis. Univariate and multivariate Cox regression analyses were applied to screen survival-associated IRGs. Then, GC patients were separated into high- and low-risk groups based on the median risk score. Furthermore, a nomogram was constructed based on the TCGA dataset. The prognostic value of the risk signature model was evaluated by Kaplan-Meier curve, receiver operating characteristic (ROC), Harrell’s concordance index and calibration curves. In addition, the gene expression dataset from the Gene Expression Omnibus (GEO) was also downloaded for external validation. The relative proportions of 22 types of infiltrating immune cells in each male GC sample were evaluated using CIBERSORT.

**Results:**

A total of 276 differentially expressed IRGs were screened, including 189 up-regulated and 87 down-regulated genes. Subsequently, a seven-IRGs signature (LCN12, CCL21, RNASE2, CGB5, NRG4, AGTR1 and NPR3) was identified to be significantly associated with the overall survival (OS) of male GC patients. Survival analysis indicated that patients in the high-risk group exhibited a poor clinical outcome. The results of multivariate analysis revealed that the risk score was an independent prognostic factor. The established nomogram could be used to evaluate the prognosis of individual male GC patients. Further analysis showed that the prognostic model had excellent predictive performance in both TCGA and validated cohorts. Besides, the results of tumour-infiltrating immune cell analysis indicated that the seven-IRGs signature could reflect the status of the tumour immune microenvironment.

**Conclusions:**

Our study developed a novel seven-IRGs risk signature for individualized survival prediction of male GC patients.

## Background

Epidemiological evidence indicates that the incidence of gastric cancer (GC) in males is nearly two times of that in females [1]. Moreover, male patients with GC always have a higher tumour-node-metastasis (TNM) stage and a poorer prognosis than their female counterparts [2]. Recently, it has been strongly recommended that sex be examined as a biological variable in future cancer research due to its substantial influence on the occurrence and development of tumours [3]. Existing widely utilized models or biomarkers may not properly predict prognosis for male GC patients. Hence, it is urgently necessary to identify novel biomarkers for survival prediction in male GC patients.

As one of the fundamental hallmarks of cancer [4], evading immune surveillance has been increasingly appreciated in recent years. The immunotherapy targeting immune checkpoint has achieved impressive success in the treatment of several tumours [5, 6]. Growing evidence indicates that tumour-infiltrating immune cells play an important role in cancer initiation and progression and have been proposed to be valuable for the diagnosis and prognosis of tumours [7–9]. In addition, immune-related genes (IRGs) within the tumour microenvironment (TME) also represent tremendous potential value in serving as prognostic biomarkers [10, 11]. Recent studies have integrated IRG expression profiles with clinical information to gain insight into the potential clinical utility of IRGs in risk stratification and survival prediction in several tumours [12–15]. In terms of the prognostic value of IRGs in GC, Jiang et al. proposed a 16-IRGs signature that could serve as a reliable prognostic tool for overall survival (OS) [16]. Nevertheless, IRGs prognostic models for male GC patients have not been reported.

This study is the first to identify novel IRGs associated with OS. We proposed an individualized prognostic model for male GC patients by using IRGs expression profiles and clinical data from The Cancer Genome Atlas (TCGA). Then, we integrated the prognostic model and clinical pathological parameters to establish a nomogram for predicting outcome. The prognostic value of IRGs signature was further validated in GSE15460 from the Gene Expression Omnibus (GEO) database. In addition, CIBERSORT was applied to elucidate the correlation between the risk signature and the abundances of infiltrative immune cells in male GC patients. We aim to provide a new insight into predicting prognosis for male GC patients.

## Materials and methods

### Online databases

The RNA-Seq data and clinical information of male GC patients were obtained from TCGA database (https://tcga-data.nci.nih.gov/tcga/). A total of 2498 IRGs were obtained from the Immunology Database and Analysis Portal (ImmPort) database (https://www.immport.org/home) [17]. We also downloaded gene expression profiles and clinical data of GSE15460 from GEO (http://www.ncbi.nlm.nih.gov/geo/) for validation dataset.

### Differentially expressed IRGs and enrichment analysis

The differential expression of IRGs between male GC and their non-tumour counterparts was identified using package “limma” in R software [18], with the cut-off value of |log2 fold change (FC)| > 1 and false discovery rate (FDR) < 0.05. Gene Ontology (GO) and Kyoto Encyclopedia of Genes and Genomes (KEGG) enrichment analyses were conducted using the R software “clusterprofiler” package to explore potential molecular mechanisms of the differentially expressed IRGs. The “ggplot2” and “GOplot” packages in R were used for visualization of GO and KEGG enrichment analysis results.

### Establishment of the prognostic IRGs signature

To improve the predictive accuracy of IRGs signature, only male GC patients with a follow-up time of more than 60 days were included in our study. Univariate and multivariate Cox regression analysis were performed to search for OS-related IRGs using “survival” package in R. The risk score of each male GC patient was calculated with the following formula: risk score = expression level of IRG_1_ × β_1_ + expression level of IRG_2_ × β_2_ +…+ expression level of IRG_n_ × β_n_; where β is the coefficient calculated by the multivariate Cox regression model [19]. Subsequently, male GC patients were divided into high- and low-risk groups according to the median risk score. The time-dependent receiver operating characteristic (ROC) curve was constructed to evaluate the predictive value of the prognostic IRGs signature for OS using the R software package “survivalROC”. By using the R package “survival”, the Kaplan–Meier survival curve was drawn to estimate the OS difference in the high- and low-risk groups, with statistical significance evaluated by the log-rank test. Moreover, to determine whether the prognostic IRGs signature could be an independent predictor of OS in male GC patients, univariate and multivariate Cox regression analyses were performed. Age, grade, stage, T stage, N stage, M stage, and risk score were employed as covariates.

### Construction of a prognostic nomogram

We formulated a nomogram on the basis of risk score and clinical parameters to assess the probability of 1-, 3-, and 5-year OS for male GC patients using the “rms,” “foreign,” and “survival” packages in R. The concordance index (C-index) was calculated to assess the performance of the prognostic nomogram. Calibration plots were also drawn to estimate the consistency between actual and predicted survival.

### External validation of IRGs signature

The risk score for each enrolled patient in the validation dataset was calculated with the same constructed formula based on the prognostic IRGs signature model. Similarly, male GC patients were divided into high- and low-risk groups according to the median risk score. Then, Kaplan–Meier curves for the high- and low-risk groups combined with log-rank test were used to assess the predictive value of the prognostic IRGs signature. Survival ROC curves were applied to assess the predictive power of the model. In addition, the calibration plots for survival probability at 3- or 5-year were generated to evaluate the prognostic accuracy of the nomogram.

### Tumour‐infiltrating immune cell analysis

CIBERSORT, a newly developed deconvolution algorithm [20], was utilized to determine the relative proportions of 22 types of infiltrating immune cells in each male GC sample. Subsequently, a Wilcoxon rank-sum test was applied to evaluate the difference in the abundance of immune cells between the high-and low-risk groups, and illustrated by the “fmsb” packages in R.

### Statistical analysis

The statistical analyses were performed using R software (version 3.6.3) and GraphPad Prism 6 (GraphPad Software Inc., USA). Kaplan–Meier curves and the log-rank test were applied to assess the statistical significance of the survival rates between the high- and low-risk groups. Univariate and multivariate Cox regression analyses were performed to evaluate significant prognostic factors. The Wilcoxon rank-sum test was used to test the statistical significance between high- and low-risk groups. Data were presented as mean ± SD and *P* < 0.05 was considered significant.

## Results

### Identification and enrichment analysis of differentially expressed IRGs

**Fig. 1:**
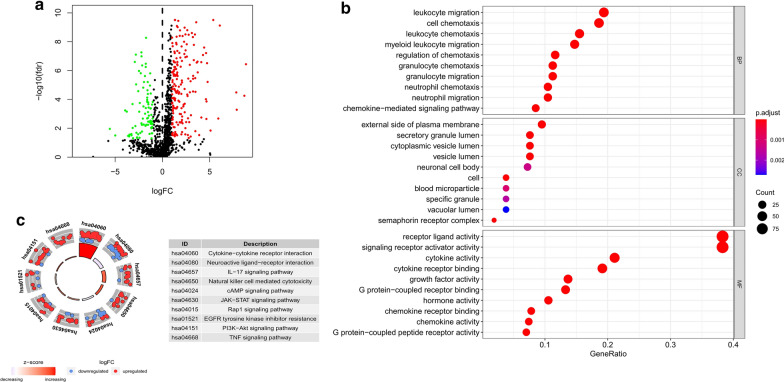
Identification and functional enrichment analysis of differentially expressed IRGs. **a** Volcano plot revealed 276 differentially expressed IRGs which including 87 downregulated (green dots) and 189 upregulated (red dots). **b **The results of GO enrichment analysis was shown by bubble chart. **c **KEGG pathways enriched in the differentially expressed IRGs

The RNA-Seq data from TCGA consisted of 263 cases, including 241 male GC cases and 22 male non-tumour cases. Compared to male non-tumour tissues, a total of 276 differentially expressed IRGs including 87 downregulated and 189 upregulated were screened out, with the cut-off criteria of |log2 fold change (FC)| > 1 and false discovery rate (FDR) < 0.05 (Fig. 1a).

Additionally, to further the investigate potential functions of the differentially expressed IRGs in male GC patients, GO and KEGG analyses were performed in R software. As shown in Fig. 1b, the results of GO enrichment analysis indicated that these IRGs can be significantly enriched in several important immune responses, including cell chemotaxis, leukocyte chemotaxis, myeloid leukocyte migration, leukocyte migration, granulocyte chemotaxis, neutrophil chemotaxis, granulocyte migration and neutrophil migration. KEGG analysis highlighted that the differentially expressed IRGs were mainly enriched in immune responses and tumour-related signalling pathways (Fig. 1c).

### Identification of OS-related IRGs

**Fig. 2:**
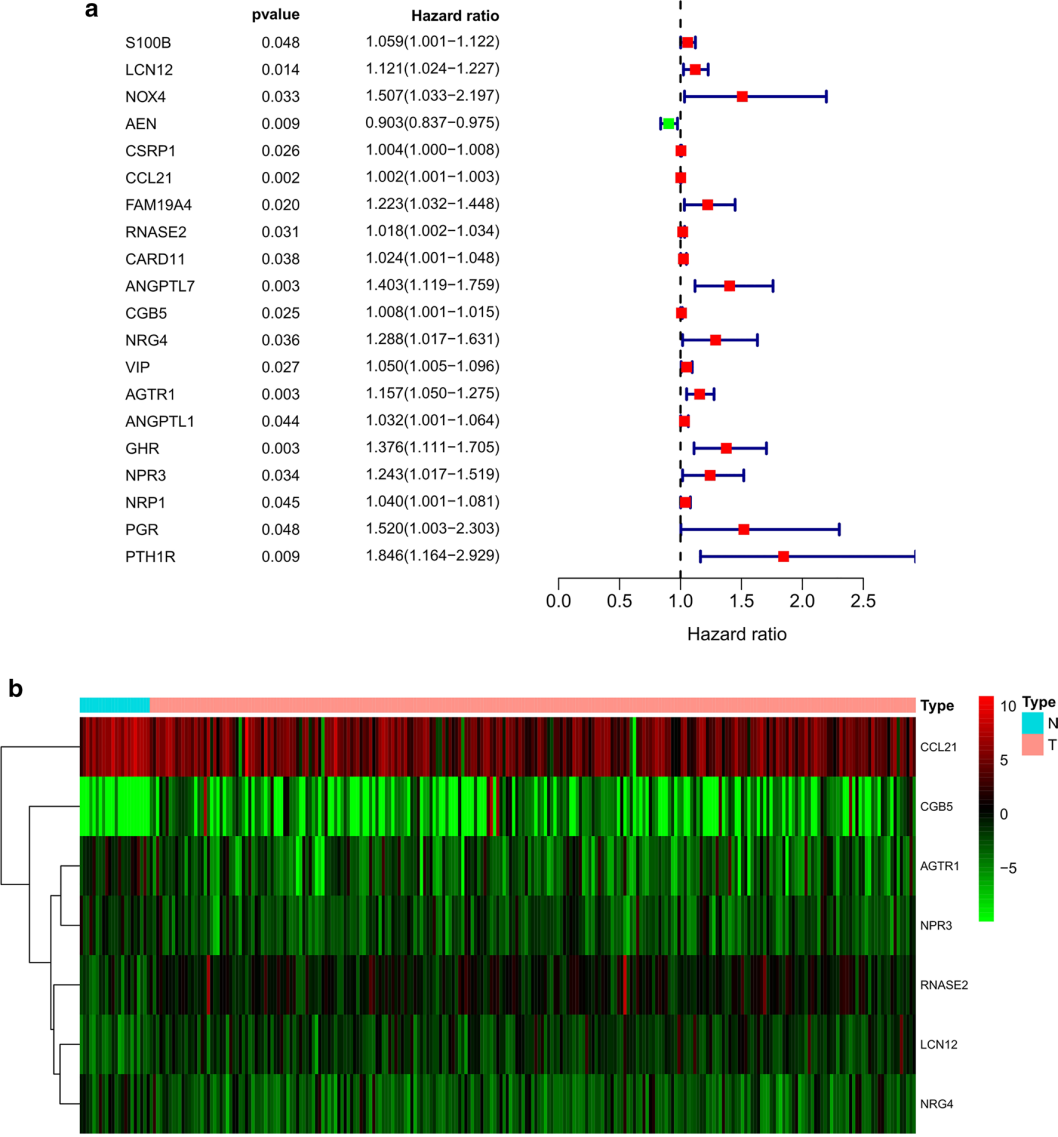
Identification of survival-associated IRGs. **a** Forrest plot of the IRGs associated with male GC survival based on the univariate Cox regression analysis. **b **Heatmap of the seven-IRGs expression profiles between male GC and non-tumour tissues

A total of 20 differentially expressed IRGs were identified to be significantly associated with the OS of male GC patients based on the results of univariate Cox regression analysis (Fig. 2a). Then, by performing multivariate Cox regression analysis, a prognostic signature consisting of seven IRGs (LCN12, CCL21, RNASE2, CGB5, NRG4, AGTR1 and NPR3) was selected to construct a prediction model (Table 1). All the seven IRGs were associated with high risk with hazard ratios (HRs) > 1. Among these IRGs, three genes (LCN12, RNASE2, and CGB5) were upregulated and four genes (CCL21, NRG4, AGTR1, and NPR3) were downregulated in male GC tissues compared to the normal tissues based on the TCGA dataset (Fig. 2b).

**Table 1:** The detail information of the seven-IRGs risk signature based on multivariate Cox regression analysis

Gene	Full name	Coefficient	HR	*P* value
LCN12	Lipocalin 12	0.13933	1.14950	0.00114
CCL21	Chemokine (C-C motif) ligand 21	0.00181	1.00181	0.00263
RNASE2	Ribonuclease a family member 2	0.01554	1.01566	0.05666
CGB5	Chorionic gonadotropin subunit beta 5	0.01036	1.01041	0.00464
NRG4	Neuregulin 4	0.34521	1.41229	0.00552
AGTR1	Angiotensin II receptor type 1	0.14512	1.15618	0.00545
NPR3	Natriuretic peptide receptor 3	0.23201	1.26113	0.03554

### Establishment of the seven-IRGs risk signature

**Fig. 3:**
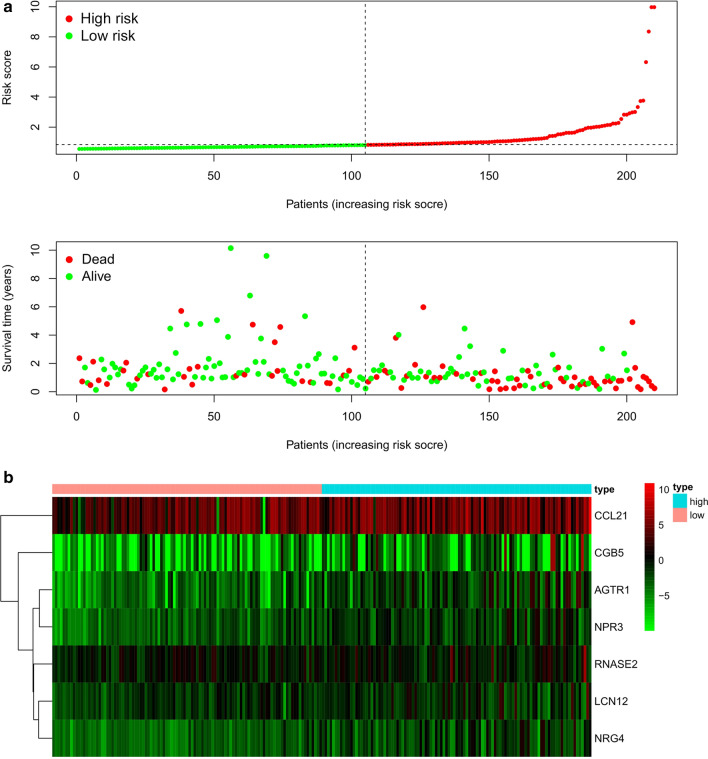
Characteristics of the seven-IRGs signature in the TCGA dataset. **a** The distribution of risk score and the survival status of male GC patients. The dotted line is the optimal cut-off value for dividing male GC patients into high- and low-risk groups. **b **Heatmap of the seven-IRGs expression profiles between the high- and low-risk groups

Subsequently, we constructed a prognostic model on the basis of the seven-IRGs. The risk score of each male GC patient was calculated as follows: risk score = expression level of LCN12 × 0.13933 + expression level of CCL21 × 0.00181 + expression level of RNASE2 × 0.01554 + expression level of CGB5 × 0.01036 + expression level of NRG4 × 0.34521 + expression level of AGTR1 × 0.14512 + expression level of NPR3 × 0.23201. The male GC patients were classified into high- and low-risk groups according to the median risk score (Additional file 1). The distribution of risk scores and the survival status of male GC patients were displayed in Fig. 3a. In addition, the heatmap revealed the differentially expressed levels of the seven-IRGs in the high- and low-risk groups (Fig. 3b).

**Fig. 4:**
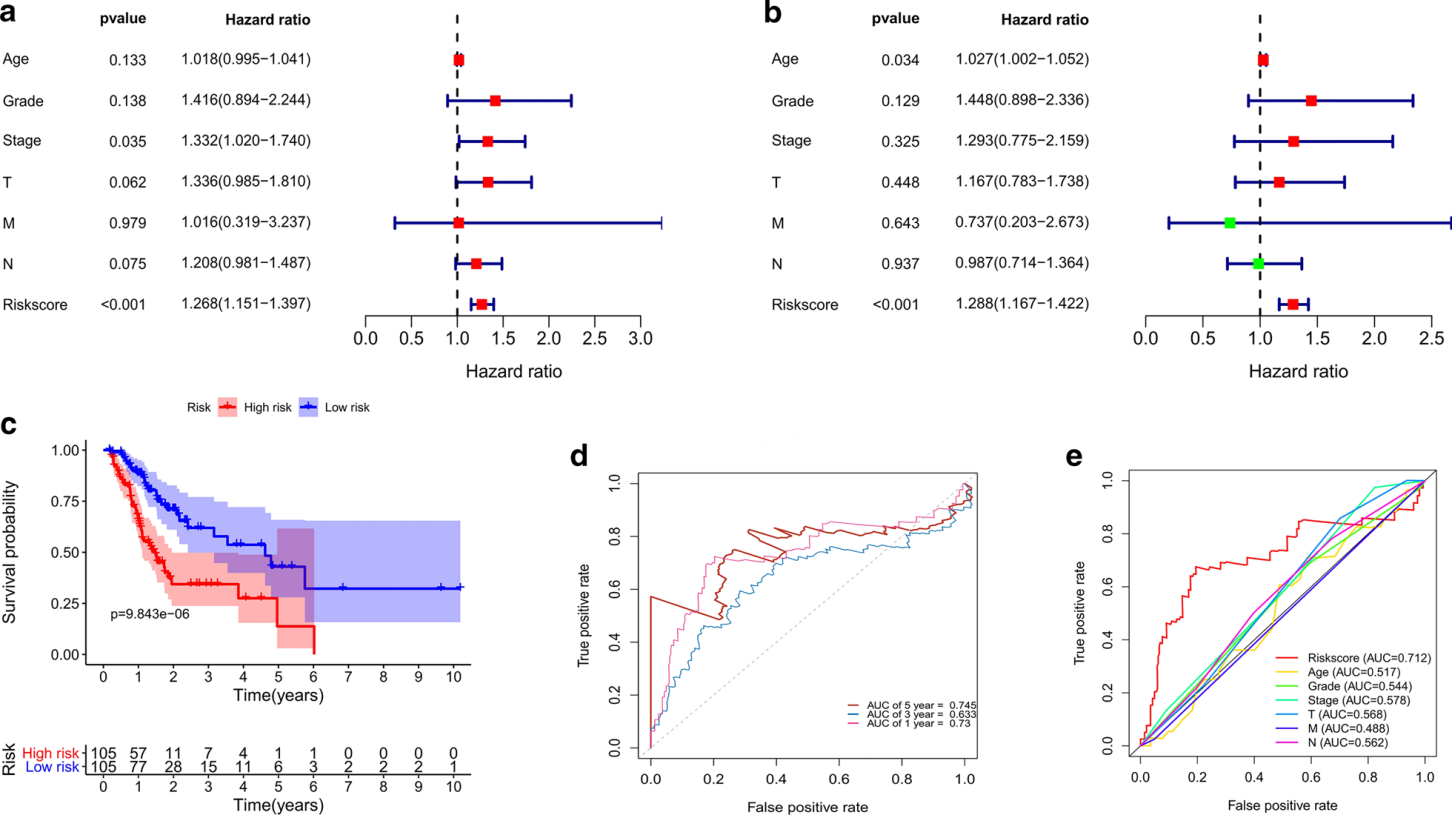
The prognostic value of the seven-IRGs signature in the TCGA dataset. **a**, **b **Univariate and multivariate Cox regression analysis showed that the risk score was significantly correlated with OS and could be an independent prognostic indicator. **c **Kaplan–Meier survival curve demonstrated that male GC patients in the high-risk group had a shorter survival time than those in the low-risk group. **d** Time-dependent ROC curves analysis of the seven-IRGs signature for 1-, 3-, and 5-year OS probability in the TCGA dataset. **e **ROC curves of the risk score and other clinical parameters with AUC scores

Next, the prognostic value of the risk score was evaluated. Univariate Cox regression analysis showed that the risk score (*P* < 0.001, HR = 1.268, 95 %CI 1.151–1.397) was significantly correlated with the OS of male GC patients (Fig. 4a). Notably, as shown in Fig. 4b, the risk score could be an independent prognostic indicator (*P* < 0.001, HR = 1.288, 95 %CI 1.167–1.422). The Kaplan–Meier curve demonstrated that male GC patients with high risk scores had a shorter survival time than those with low risk scores (log-rank *P* < 0.001, Fig. 4c). The areas under the curve (AUCs) for the risk score at 1-, 3- and 5-year in predicting OS were 0.73, 0.633 and 0.745, respectively (Fig. 4d). Moreover, compared to other clinical parameters, the risk score had the highest performance in the survival prediction of male GC patients (AUC = 0.712, Fig. 4e). Taken together, the above results indicated that the risk score performed well at predicting OS in the TCGA dataset.

### Construction of the prognostic nomogram

**Fig. 5:**
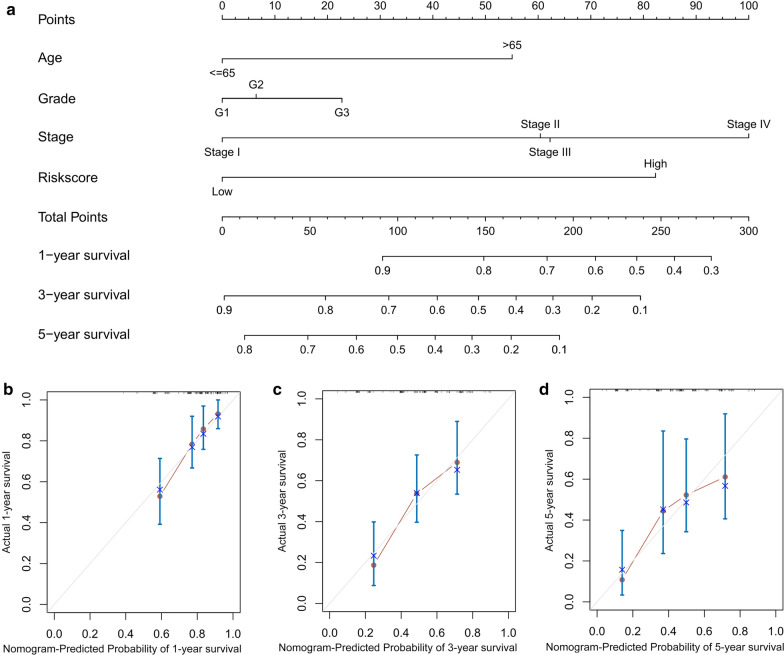
Construction of the nomogram for predicting the prognosis probability in the TCGA dataset. **a **Prognostic nomogram to predict the OS of male GC patients. **b–d** Calibration curves of the nomogram for predicting survival at 1-, 3-, and 5-years in the TCGA dataset. The nomogram-predicted probability is plotted on the x-axis and the actual survival is plotted on the y-axis

To better predict the prognosis of male GC patients, we established a nomogram to predict the OS probability at 1-, 3- and 5-years (Fig. 5a). The variables of age, grade, stage and risk score were included in the prediction model. The C-index of the nomogram was 0.695 (95 % CI 0.632–0.759). As shown in Fig. 5b, the calibration plots demonstrated favourable agreement between predicted probabilities from the nomogram and the observed outcomes. Collectively, these results implied that the nomogram had good reliability in predicting survival for male GC patients.

### External validation of the seven-IRGs prognostic signature

**Fig. 6:**
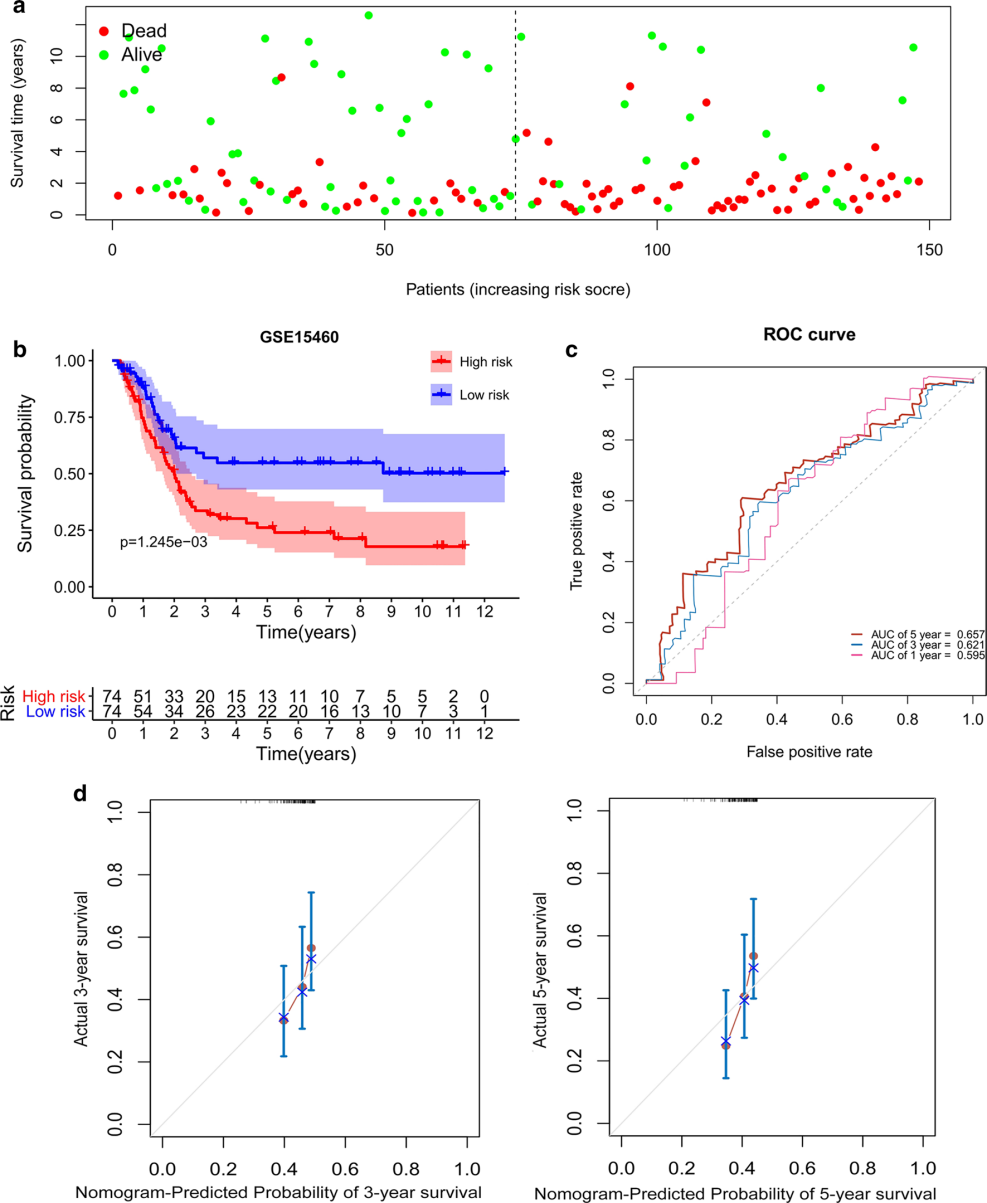
External validation of the seven-IRGs prognostic risk model. **a **The survival status and time of male GC patients distributed by risk score in GSE15460. The dotted line is the optimal cut-off value for dividing male GC patients into high- and low-risk groups. **b **Kaplan-Meier survival curve of male GC patients in the high- and low-risk groups in GSE15460. **c** Time-dependent ROC curves analysis of the seven-IRGs signature for 1-, 3-, and 5-year OS probability in GSE15460. **d **Calibration curves of the nomogram for predicting survival at 3-, and 5-years in GSE15460. The nomogram-predicted probability is plotted on the x-axis and the actual survival is plotted on the y-axis

To further examine the performance of the seven IRGs-based model, the gene expression data and survival outcomes from GSE15460 were used for external validation. We calculated the risk score with the same formula for each male GC patient, and then divided them into high- and low‐risk groups according to the median risk score (Additional file 2). Compared with patients in the low‐risk group, male GC patients with high risk scores suffered significantly more survival risks (Fig. 6a). Similar to the abovementioned findings, male GC patients in the high-risk group were associated with poor survival outcomes (log-rank *P* = 0.001, Fig. 6b). As presented in Fig. 6c, the AUCs of 1-, 3- and 5-year in predicting OS were 0.595, 0.621 and 0.657, respectively. Furthermore, the calibration plots indicated quite good agreement between prediction and observation for the 3- and 5-year OS probabilities of the patients (Fig. 6d).

### Associations of the risk signature with tumour‐infiltrating immune cells

**Fig. 7:**
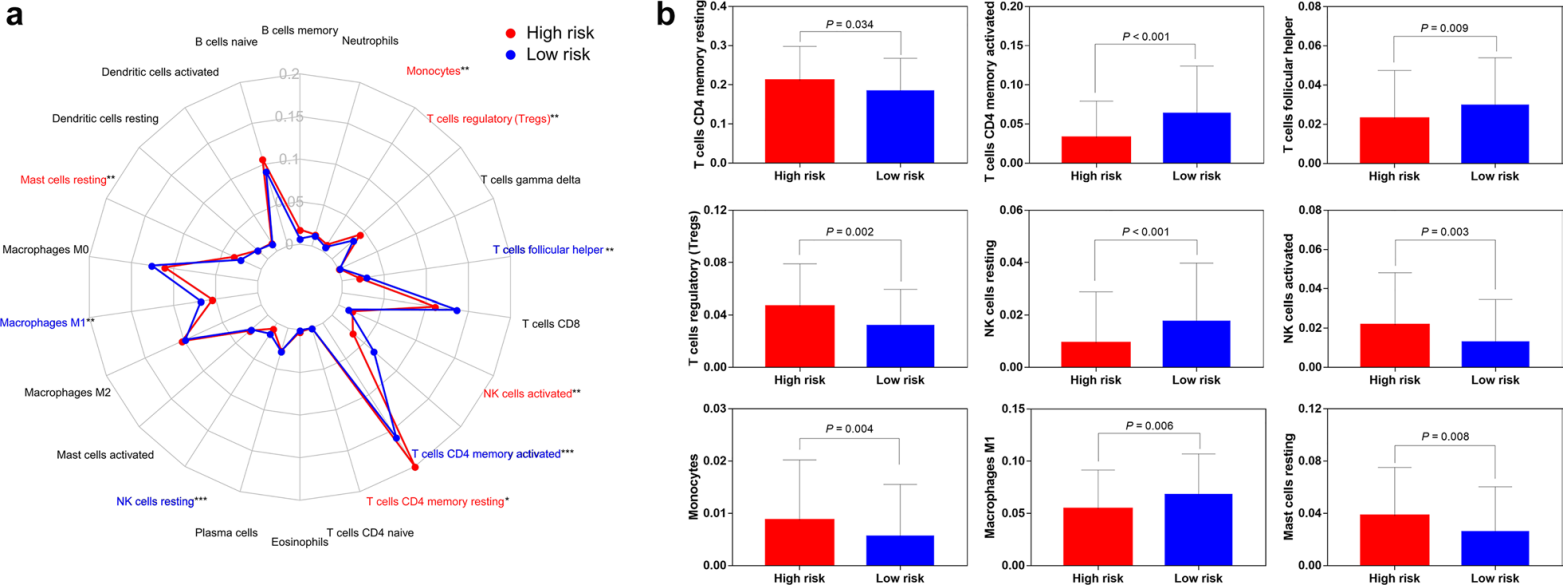
Associations of the seven-IRGs signature with tumour-infiltrating immune cells in TCGA dataset. **a **Differential distribution of 22 infiltrating immune cell types between high- and low-risk groups. **b** The bar chart exhibited the significant infiltrating difference of several immune cells in the high- and low-risk groups. ^*^P < 0.05, ^**^P < 0.01, ^***^P < 0.001

To investigate distinct patterns of immune infiltration, we used CIBERSORT algorithm to estimate the composition of 22 infiltrating immune cells types in each male GC sample. As presented by the radar plot in Fig. 7a, the abundance of the 22 infiltrative immune cells was significantly different between high- and low-risk groups. Specifically, the infiltration levels of resting memory CD4 T cells (*P* = 0.034), activated NK cells (*P* = 0.003), regulatory T cells (Tregs) (*P* = 0.002), monocytes (*P* = 0.004) and resting mast cells (*P* = 0.008) were significantly higher in the high-risk group than in the low-risk group, whereas the infiltration levels of activated memory CD4 T cells (*P* < 0.001), resting NK cells (*P* < 0.001), follicular helper T cells (*P* = 0.009) and M1 macrophages (*P* = 0.006) were the opposite (Fig. 7b).

## Discussion

Although the morbidity and mortality of GC have declined over the past decade [21], we still face many problems and challenges in the screening and treatment of GC. Because of sex differences and tumour heterogeneity, even patients with the same pathologic stage may have considerable differences in survival, indicating that prognosis cannot be accurately determined based on the current staging system alone [22]. Epidemiological data and studies have pointed out that males suffer a higher risk and poorer prognosis than females in many types of cancer [23, 24]. Finding new predictors with good prognostic value for male GC patients is urgently needed, which is also in line with the development of precision medicine.

The role of the immune system in cancer is twofold: it not only suppresses tumour growth but also promotes tumour progression [25]. Because of the complexity of immune response and tumour biology, it is difficult to predict the survival of patients only by a single biomarker. In this study, we first screened 276 differentially expressed IRGs between male GC and non-tumour tissues. Bioinformatics enrichment analysis demonstrated that these IRGs were mainly related to immune responses and several tumour-related signalling pathways. Then, based on the results of univariate and multivariate Cox regression analysis, an immune related risk score model for male GC patients, which is composed of seven differentially expressed IRGs, was constructed using TCGA dataset. Male GC patients with high risk scores had significantly poorer OS than those with low risk scores. The risk score could be an independent prognostic indicator for male GC patients. Moreover, the prognostic value of IRGs signature was further validated in an external dataset.

Among the seven IRGs, four IRGs (CCL21, CGB5, NRG4 and AGTR1) have been reported to be involved in the development and progression of GC. Concretely speaking, Tang et al. reported that CCL21 is overexpressed in Epstein–Barr virus-associated GC and protects CD8^+^CCR7^+^ T lymphocytes from apoptosis via the mitochondria-mediated pathway [26]. In addition, high expression of CCL21 was related to lymph node metastasis and poor prognosis in GC patients [27]. CGB5 was found to be overexpressed in most GC tissues and was considered as an independent predictor of OS and recurrence-free survival for GC patients [28]. As the specific ligand of HER4, the role of NRG4 in GC remains controversial. A previous study demonstrated both HER4 and NRG4 were downregulated in GC tissues compared to matched normal tissues [29]. In contrast, another study indicated that HER4 was overexpression in GC, but not associated with survival [30]. Notably, the NRG4-HER4 axis might also play an important role in the proliferation of malignant lymphoma cells in the gastrointestinal tract [31]. In GC, the utility of AGTR1 inhibitor significantly suppresses GC cell proliferation and stromal fibrosis [32]. It has been proven that AGTR1 could enhance malignant phenotype of several cancer cells to promote tumour progression [33–35]. The roles of RNASE2 and NPR3 in GC have not been reported. A recent study figured out that RNASE2 could be a valuable prognostic predictor in clear cell renal cell carcinoma [36]. Moreover, the expression of RNASE2 was significantly upregulated in childhood acute lymphoblastic leukaemia [37]. Li et al. reported that MRCCAT1 promotes metastasis of clear cell renal cell carcinoma via inhibiting NPR3 expression [38]. However, in hepatocellular carcinoma, FENDRR promoted cancer cells apoptosis by targeting miR-362-5p via stimulating NPR3 expression [39]. In addition, NPR3 upregulation could also promote the proliferation of colorectal cancer cells [40]. There are few studies about LCN12, and its role in cancer has not yet been elucidated. LCN12 belongs to the lipocalin family of proteins, which is associated with male reproduction and immune response [41, 42].

In addition, a nomogram combined with our risk signature model and clinicopathological parameters was developed to predict the 1-, 3- and 5-year OS of male GC patients. To our knowledge, the seven-IRGs signature related prognostic model and nomogram have not been reported to date. This nomogram could provide an intuitive visual presentation of individualized survival prediction for both doctors and patients.

Finally, given the critical role of tumour-infiltrating immune cells in tumour progression, the difference of the composition of 22 infiltrating immune cells types in the high- and low-risk groups was analysed. We found that the percentages of resting memory CD4 T cells, activated NK cells, Tregs, monocytes and resting mast cells were significantly higher in the high-risk group, while activated memory CD4 T cells, resting NK cells, follicular helper T cells and M1 macrophages were mainly enriched in the low-risk group. This result was basically consistent with previous studies. For instance, a recent study revealed that resting memory CD4 T cells was one of the most abundant tumour-infiltrating immune cells in GC samples; in addition, the infiltration levels of activated memory CD4 T cells was positively correlated with a favourable prognosis for GC patients [43]. It was reported that GC patients with high infiltration of FOXP3(+) Tregs exhibited a lower OS rate and a poor outcome [44]. In the TME, macrophages could display antitumour M1 and protumour M2 phenotypes, and high density of M1 macrophages was associated with better OS in GC patients [45, 46]. Follicular helper T cells have been discovered in tertiary lymphoid structures of several tumours, suggesting that they might play a vital role in the generation of effective and durable antitumour immune responses [47–49]. Additionally, mast cells and monocytes act as proinflammatory, angiogenic, lymphangiogenic and immunomodulatory mediator in GC progression [50]. Indeed, we also noticed that tumour-associated monocytes/macrophages could impair NK cells function through TGFβ1 to promote GC immune escape [51]. Hence, the seven-IRGs risk signature might reflect a changing TME for male GC patients in the high-risk group. Collectively, these results could at least partially explain the poorer outcome for the high-risk group.

However, the current study has several limitations that should be taken into consideration. First, since our study object was only male GC patients, the enrolled and validated sample size was relatively small. Second, more large-scale, multicenter and prospective clinical cohorts are needed to verify the predictive value of the seven-IRGs signature. Third, further experimental studies may also be needed to elucidate underlying the molecular mechanisms of the seven-IRGs in GC.

## Conclusions

In summary, we identified a seven-IRGs signature and established a reliable prognostic nomogram model for OS prediction in male GC patients.

## Supplementary Information

**Additional file 1.** The detail of risk score for each male GC patient in TCGA dataset.

**Additional file 2.** The detail of risk score for each male GC patient in GSE15460.

## Data Availability

Not applicable.

## Abbreviations

GC: Gastric cancer
IRGs: Immune-related genes
TME: Tumour microenvironment
GEO: Gene Expression Omnibus
TCGA: The Cancer Genome Atlas
ROC: Receiver operating characteristic
GO: Gene ontology
KEGG: Kyoto Encyclopedia of Genes and Genomes
HR: Hazard ratios
AUC: Area under the curve
OS: Overall survival
